# Brain-specific angiogenesis inhibitor 1 is expressed in the Myo/Nog cell lineage

**DOI:** 10.1371/journal.pone.0234792

**Published:** 2020-07-02

**Authors:** Jacquelyn Gerhart, Jessica Bowers, Lindsay Gugerty, Colby Gerhart, Mark Martin, Fathma Abdalla, Arturo Bravo-Nuevo, Jonathan Tabb Sullivan, Rebecca Rimkunas, Amie Albertus, Lou Casta, Lori Getts, Robert Getts, Mindy George-Weinstein

**Affiliations:** 1 Division of Research, Philadelphia College of Osteopathic Medicine, Philadelphia, PA, United States of America; 2 Genisphere, LLC, Hatfield, PA, United States of America; 3 Integral Molecular, Philadelphia, PA, United States of America; Medical College of Wisconsin, UNITED STATES

## Abstract

The Myo/Nog cell lineage was discovered in the chick embryo and is also present in adult mammalian tissues. The cells are named for their expression of mRNA for the skeletal muscle specific transcription factor MyoD and bone morphogenetic protein inhibitor Noggin. A third marker for Myo/Nog cells is the cell surface molecule recognized by the G8 monoclonal antibody (mAb). G8 has been used to detect, track, isolate and kill Myo/Nog cells. In this study, we screened a membrane proteome array for the target of the G8 mAb. The array consisted of >5,000 molecules, each synthesized in their native confirmation with appropriate post-translational modifications in a single clone of HEK-293T cells. G8 mAb binding to the clone expressing brain-specific angiogenesis inhibitor 1 (BAI1) was detected by flow cytometry, re-verified by sequencing and validated by transfection with the plasmid construct for BAI1. Further validation of the G8 target was provided by enzyme-linked immunosorbent assay. The G8 epitope was identified by screening a high-throughput, site directed mutagenesis library designed to cover 95–100% of the 954 amino acids of the extracellular domain of the BAI1 protein. The G8 mAb binds within the third thrombospondin repeat of the extracellular domain of human BAI1. Immunofluorescence localization experiments revealed that G8 and a commercially available BAI1 mAb co-localize to the subpopulation of Myo/Nog cells in the skin, eyes and brain. Expression of the multi-functional BAI1 protein in Myo/Nog cells introduces new possibilities for the roles of Myo/Nog cells in normal and diseased tissues.

## Introduction

The Myo/Nog lineage was discovered in the epiblast of the chick embryo blastocyst [1–3]. The cells were identified by their co-expression of mRNA for the skeletal muscle specific transcription factor MyoD and bone morphogenetic protein (BMP) inhibitor Noggin [1–3]. A third marker of Myo/Nog cells is the cell surface molecule recognized by the G8 monoclonal antibody (mAb) [2–4]. This mAb was used to track Myo/Nog cells from the epiblast into tissues and organs throughout the embryo [3, 5]. *In vitro* and *in vivo* analyses of Myo/Nog cells purified by fluorescence activated and magnetic cell sorting of G8 bound cells revealed their stable expression of Noggin and commitment to the skeletal muscle lineage regardless of their environment [2, 4, 5].

The G8 mAb also has used to specifically target Myo/Nog cells for depletion by complement mediated cell lysis [3, 6–8]. Embryos depleted of Myo/Nog cells lacked skeletal muscle and exhibited severe malformations of the central nervous system, eyes, face and ventral body wall as a result of hyperactive BMP signaling [3, 6, 7]. These studies demonstrated that release of Noggin by Myo/Nog cells is indispensable for normal embryonic development.

Expression of MyoD in Myo/Nog cells is the hallmark of their ability to differentiate into multinucleated skeletal myofibers and myofibroblsts [8–12]. G8 mAb targeted elimination of Myo/Nog cells in human lens tissue prevented the emergence of myofibroblasts [12, 13]. Injection of G8 conjugated to 3DNA intercalated with Doxorubicin into the rabbit lens during cataract surgery nearly eliminated myofibroblasts and their contractions that contribute to the formation of secondary cataracts [10]. [10].

The above-mentioned studies illustrate the indispensable utility of the G8 mAb for identifying, tracking, isolating and killing Myo/Nog cells. However, identification of the target of G8 has remained elusive with standard assays of antibody/protein interactions. In this study, we utilized relatively new technology to identify the G8 target that involved screening a membrane proteome array. The G8 mAb bound to brain-specific angiogenesis inhibitor 1 (BAI1). A shotgun mutagenesis approach was used to localize the G8 epitope to the third thrombospondin repeat of BAI1’s extracellular domain. A shotgun mutagenesis approach was used to localize the G8 epitope to BAI1’s extracellular domain. Immunofluorescence localization experiments confirmed co-localization of G8 and a commercially available anti-BAI1 mAb to Myo/Nog cells in multiple tissues.

## Materials and methods

### Identification of the target of the G8 mAb

The target of the G8 mAb was identified with the Membrane Protein Array (MPA) technology platform developed by Integral Molecular (Philadelphia, PA). The MPA platform is designed to profile the specificity of antibody binding to a library of 5,300 human membrane proteins expressed in HEK-293T cells in their native confirmation with appropriate post-translational modifications (Tucker et al., 2018). After the array was transfected using lipofectamine (Invitrogen; ThermoFisher Scientific, Waltham, MA), the cells were incubated for 36 hours, detached using CellStripper (Corning; VWR, Radnor, PA) and re-formatted into a two-dimensional matrix in a new 384-well plate (Corning; VWR) by rows and columns using a JANUS Automated Workstation (PerkinElmer, Waltham, MA). Each well on the matrix plate contained 48 different overexpressed protein constituents. Each protein is represented in a unique combination of two different wells of the matrix plate, contained within a “row” pool and a “column” pool. A concentration of 30 μg/ml of the G8 mAb was used to screen the plates. Antibody binding was detected by flow cytometry using a fluorescent secondary antibody. Fluorescence readings from each experimental plate were validated with single positive clones of HEK-293T cells transfected with the construct expressing known target and negative controls (empty vector) using serial dilutions of the G8 mAb from 20–0.31 μg/ml. The resulting binding values were normalized and transformed to give a single numerical value for binding of the mAb against each target protein (normalized target binding). Non-specific fluorescence was determined to be any value below three standard deviations above noise. The experiment was repeated with a second lot of G8 mAb and every concentration of mAb was run in quadruplicates. The identity of the target was re-verified by sequencing.

Further validation of the G8 mAb target was provided by enzyme-linked immunosorbent assay (ELISA) using 1 μg/ml recombinant human BAI1 protein, amino acids 31–879, produced by the Chinese Hamster Ovary (CHO) cell line (Accession #: 014514) (4969-BA-050, R&D Systems) as the substrate. Plates were coated with substrate protein for one hour at room temperature. The G8 IgM mAb and BAI1 IgG mAb (MAB4969, R&D Systems, Minneapolis, MN) were added in 100 μl of phosphate buffered saline (Applied Biosciences, ThermoFisher Scientific) at concentrations of 0.18–1.8 μg/well and 0.08–0.2 μg/100 μl/well, respectively. Binding of G8 and the R&D BAI1 mAbs were detected with anti-mouse IgM and IgG affinity purified F(ab’)2 secondary antibodies, respectively, conjugated with horseradish peroxidase diluted 1:20,000 (Jackson ImmunoResearch, Laboratories, Inc., West Grove, PA). ELISA was performed in triplicate.

### Mapping of the G8 mAb epitope

Shotgun Mutagenesis epitope mapping was performed by Integral Molecular as described previously [14]. Briefly, a mutation library designed to cover 95–100% of the 954 amino acid residues in the extracellular domain of the BAI1 protein (UniProt O14514) was created by high-throughput, site-directed mutagenesis. Each residue was individually mutated to alanine, and alanine codons were mutated to serine. The library was arrayed in 384-well microplates and transiently transfected into HEK-293 cells. Complimentary DNA clones were sequenced to verify the mutation. Cells were incubated with G8 and the reference 5D6 anti-BAI1 mAb (Integral Molecular) at concentrations that were optimized using an independent immunofluorescence titration curve on wild type protein. The 5D6 mAb was generated by Integral Molecular as part of an effort to develop an array of antibodies to brain associated targets. 5D6 was run on the MPA and was shown to be specific to BAI1/ADGRB1. The 5D6 antibody was used in the epitope mapping study to rule out residues that are likely causing structural perturbations that non-specifically reduce binding to conformational epitopes.

MAbs were detected with an Alexa Fluor 488-conjugated secondary antibody. The mean cellular fluorescence was determined using the Intellicyt iQue flow cytometry platform. The percent of binding was compared to the reference anti-BAI1 5D6 mAb (Integral Molecular). Mutated residues were identified as being critical to the G8 epitope if binding of the G8 mAb was reduced by a minimum of 80% of that achieved with the 5D6 mAb. This counterscreen strategy facilitates exclusion of mutants that are locally misfolded or that have an expression defect. Clones were rescreened in quadruplicate to confirm the results.

### Tissue procurement

A six mm punch biopsy needle was used to collect samples of tattooed skin from human bodies donated to the Philadelphia College of Osteopathic Medicine through the Humanity Gifts Registry of Pennsylvania. Tissue was embedded in paraffin and sectioned at 10 μM.

Anterior human lens tissue was removed by capsulorhexis during cataract surgery. Procurement of tissue was carried out in accordance with the Declaration of Helsinki and approved by the Philadelphia College of Osteopathic Medicine’s Institutional Review Board (#H17-018). Written consent was obtained from all patients. Tissue was collected in Dulbecco’s modified Eagle’s medium (DMEM)/F12) containing 3 IU penicillin and 30 μg streptomycin (Gibco; ThermoFisher Scientific) and fixed in 2% paraformaldehyde within an hour of collection, or placed directly in fixative.

New Zealand white rabbits underwent cataract surgery as described previously [10]. Anesthesia was obtained with an intramuscular injection of ketamine hydrochloride (50 mg/kg) and xylazine (7 mg/Kg). One drop of topical proparacaine hydrochloride anesthetic was placed in each eye before surgery and all efforts were made to minimize suffering. The procedures adhered to the Association for Research in Vision and Ophthalmology Statement for the Use of Animals in Ophthalmic and Vision Research and approved by the Institutional Animal Care and Use Committee (IACUC) of the University of Utah. The eyes were harvested two days after surgery, fixed in 10% neutral buffered formalin, bisected coronally, and the anterior cavity was embedded in paraffin and sectioned at 10 μM.

The eyes were enucleated from male 6-month old Sprague Dawley rats after euthanasia (Philadelphia College of Osteopathic Medicine’s IACUC protocol # A17-007). They were fixed in 4% formaldehyde for 3 hours, rinsed with phosphate buffered saline, immersed in 30% sucrose overnight, embedded and frozen in OCT, and sectioned at 20 μM.

Brains were removed from 10-day old C57 mice (Philadelphia College of Osteopathic Medicine’s IACUC protocol # A17-010). The hemispheres were separated and placed in 4% formaldehyde for 24 hours prior to embedding in paraffin. Both hemispheres were sectioned at 7 μM.

### Immunofluorescence localization

Human lens explant tissue and tissue sections from human skin, rabbit and rat eyes, and mouse brains were examined for binding of the G8 IgM mAb and the R&D BAI1 IgG mAb as described previously [1, 3, 4]. The G8 mAb was generated by immunizing mice with chick embryo somite and segmental plate mesoderm cells [4]. The immunogen for the R&D BAI1 mAb was produced in Chinese hamster ovary cells expressing recombinant human BAI1 amino acids 1–879. G8 IgM mAb culture supernatant was diluted 1:20 and purified G8 IgM was used at a concentration of 3.6 μg/ml. The R&D BAI1 mAb was diluted 1:200. Double labeling also was performed with the R&D BAI1 mAb and the anti-Noggin goat polyclonal antiserum (AF719; R&D Systems), anti-MyoD IgG1 mAb (MA5-12902, ThermoFisher Scientific, Rockford, IL), anti-MyoD rabbit polyclonal antiserum (ab203383, Abcam, Cambridge, MA), anti-NeuN IgG mAb (ab104224, Abcam), anti-GFAP IgG mAb (ab10062, Abcam) and anti-Iba1 rabbit mAb (ab178846, Abcam), all diluted 1:100. The anti-Iba1 goat polyclonal antiserum (ab5076, Abcam) was diluted 1:50. The numbers of animals, tissues and tissue sections labeled with each combination of antibodies are given in Table 1. All fluorescent cells in every section were identified as double or single labeled. Quantitative analyses of the total number of cells double labeled with the R&D BAI1 mAb and antibodies to G8, Noggin and MyoD were performed on a subset of tissue sections. The numbers of single labeled cells were quantified in all sections.

**Table 1:** Tissue sources and numbers of tissue and tissue sections screened by immunofluorescence localization of antibodies.

Tissue	# subjects/animals	Antibodies: # sections or tissue pieces analyzed
Human tattooed skin	6	G8/R&D BAI1: 18
		Noggin/R&D BAI1: 12
		G8/Noggin: 25
		MyoD/BAI1: 22
		G8/MyoD: 8
		No primary antibody controls: 14
Human lens tissue	12	G8/R&D BAI1: 4
		Noggin/R&D BAI1: 4
		G8/Noggin: 4
		No primary antibody controls: 4
Rabbit anterior cavity	9	G8/R&D BAI1: 14
		Noggin/R&D BAI1: 13
		MyoD/BAI1: 18
		No primary antibody controls: 5
Rat retina	1	G8/R&D BAI1: 8
		Noggin/R&D BAI1: 4
		No primary antibody controls: 4
Mouse brain	1 brain, 2 hemispheres	G8/R&D BAI1: 26
		Noggin/R&D BAI1: 18
		G8/Iba1: 12
		G8/GFAP: 12
		G8/NeuN: 43
		No primary antibody controls: 10

Primary antibodies were tagged with AffiniPure Fab fragment subclass and species-specific secondary antibodies conjugated with Rhodamine Red and Alexa 488 (Jackson ImmunoResearch Laboratories, Inc.) diluted 1:400. The level of background fluorescence was assessed by labeling tissue sections and explants with secondary antibodies only.

### Microscopy

Tissues were analyzed with the Nikon Eclipse E800 epifluorescent microscope (Nikon Instruments Inc., Melville, NY) equipped with 10x, 60x and 100x lenses, the Evolution QE Optronics video camera and Image Pro Plus image analysis software program (Media Cybernetics, Rockville MD). Tissues also were analyzed with the Olympus Confocal Fluoview 100 microscope (Olympus Corp., Tokyo, Japan) equipped with 10x and 60x lenses and the Fluoview software program. Sections were photographed with a 60X plan apo, 1.4 numerical aperture lens. Photographs were adjusted for brightness and contrast, and figures annotated with Adobe Photoshop CC 2014 (Adobe Inc., San Jose, CA).

## Results

### The target of the G8 mAb is BAI1

**Fig 1:**
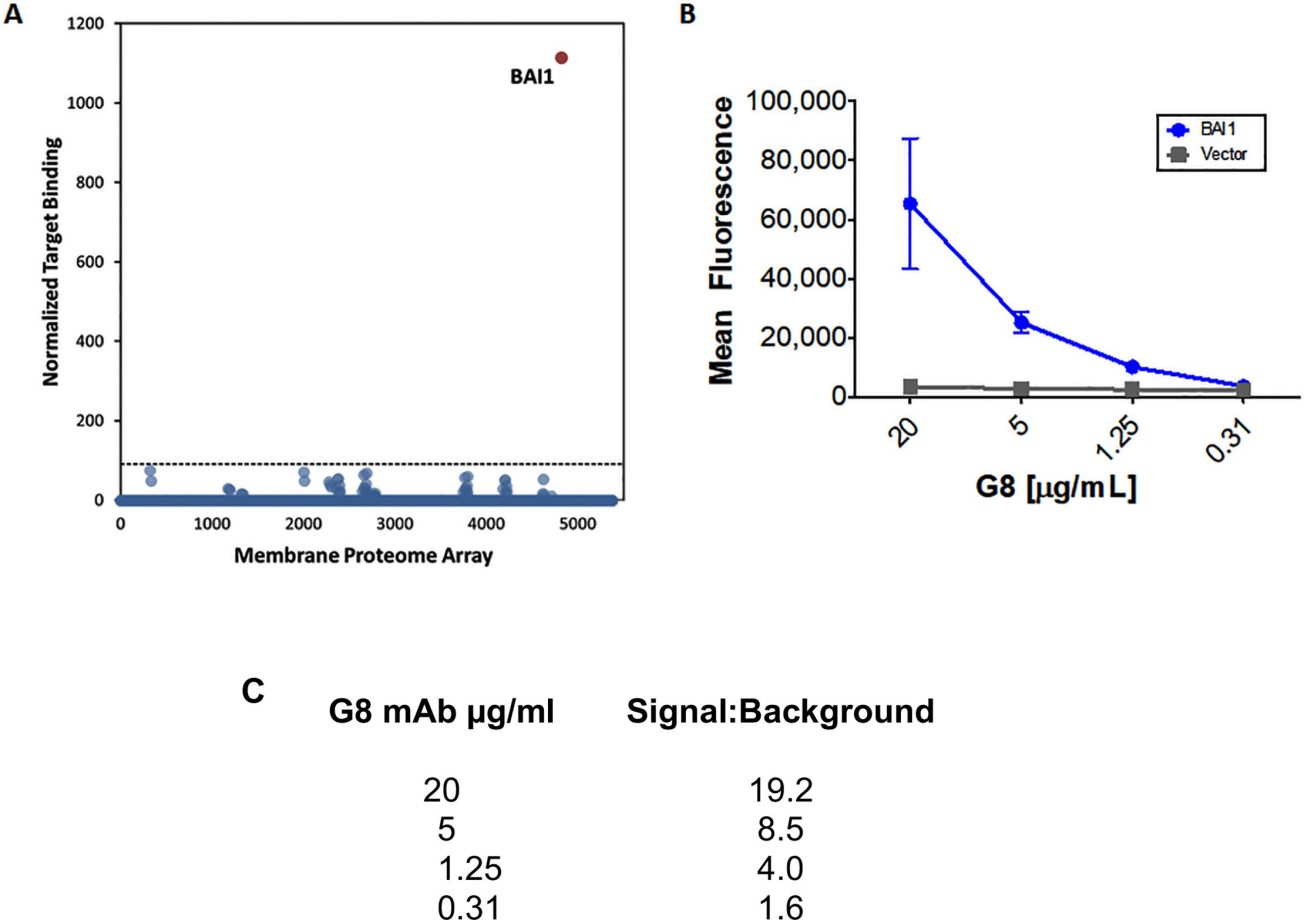
Identification of the target of the G8 mAb. The G8 mAb was used to screen clones of HEK-239T cells expressing a single human membrane protein. G8 binding was detected by flow cytometry. A. Binding values were normalized and transformed to give a single numerical value for binding of the G8 mAb against each target protein (normalized target binding). Non-specific fluorescence was determined to be any value below three standard deviations above noise. G8 binding above background occurred with the clone expressing BAI1. B. Validation of binding of the G8 mAb to BAI1 or vector alone was carried out with HEK-293T cells transfected with the plasmid construct expressing target using a serial dilution of mAb. C. Binding of different concentrations of the G8 mAb was calculated as the signal to background ratio of fluorescence from the BAI1 expressing clone to empty vector clone.

The membrane proteome array (MPA) platform (Integral Molecular) was used to identify the target of the G8 mAb. Binding of G8 at levels above background was detected in clones of HEK293T cells expressing BAI1 (Fig 1A). Validation of protein binding targets was carried out with HEK293T cells transfected with the BAI1 plasmid construct. Serial dilutions of the G8 mAb from 20–1.25 μg/ml bound to BAI1 expressing cells (Fig 1B and 1C). A second lot of the G8 mAb produced similar results (signal to background ratios of 16.1–1.7).

**Fig 2:**
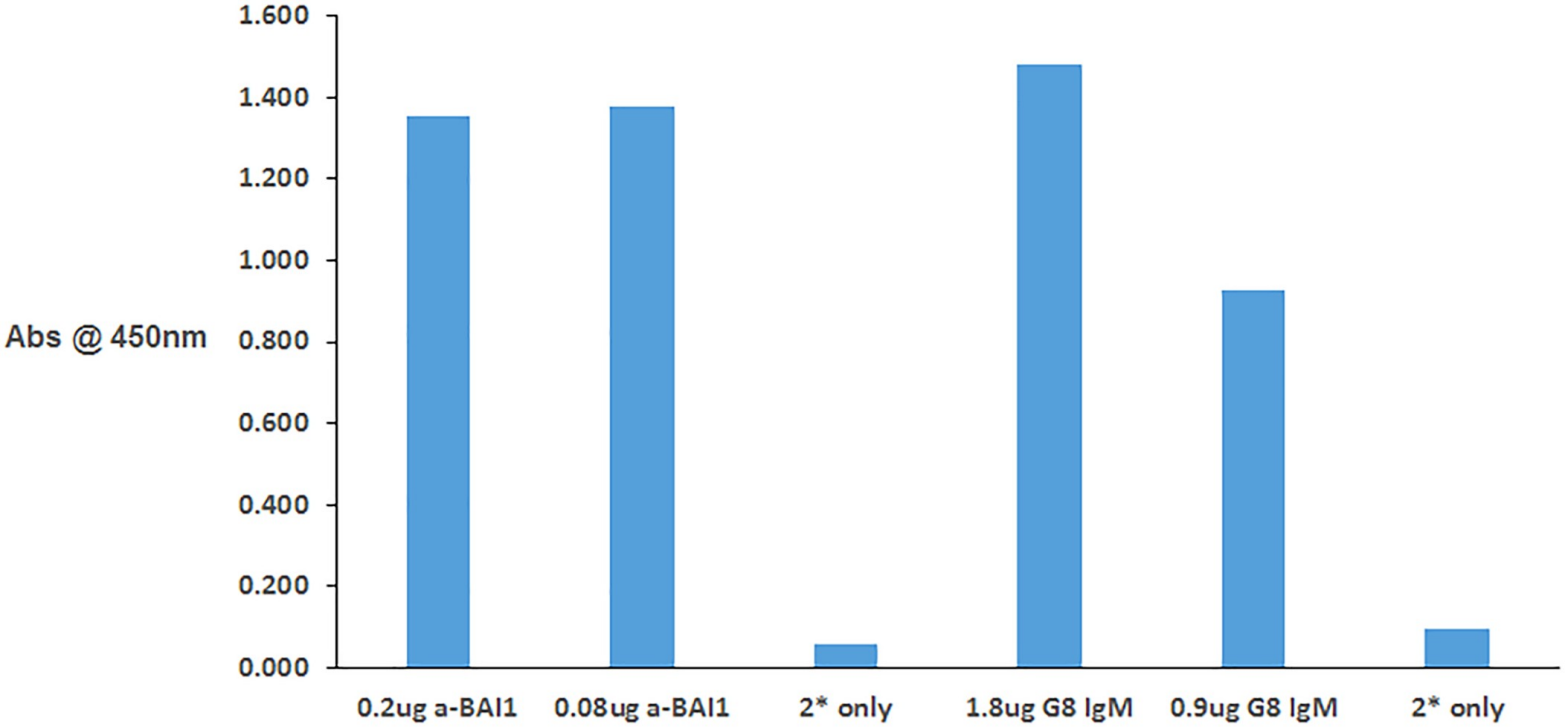
The G8 mAb binds to purified BAI1 in ELISA. Plates were coated with recombinant human BAI1 protein produced by CHO cells. Binding of the G8 IgM mAb and R&D BAI1 IgG mAb (a-BAI) was detected with anti-mouse IgM and IgG affinity purified F(ab’)2 secondary antibodies, respectively, conjugated with horseradish peroxidase. Both mAbs bound to BAI1 protein.

Further validation of the G8 mAb target was obtained by ELISA. Both G8 and anti-BAI1 mAb from R&D Systems (R&D BAI1) bound to a substrate of purified BAI1 protein containing amino acids 31–879 located within the extracellular domain (Fig 2).

### The G8 epitope is located in the third thrombospondin repeat of BAI1

**Fig 3:**
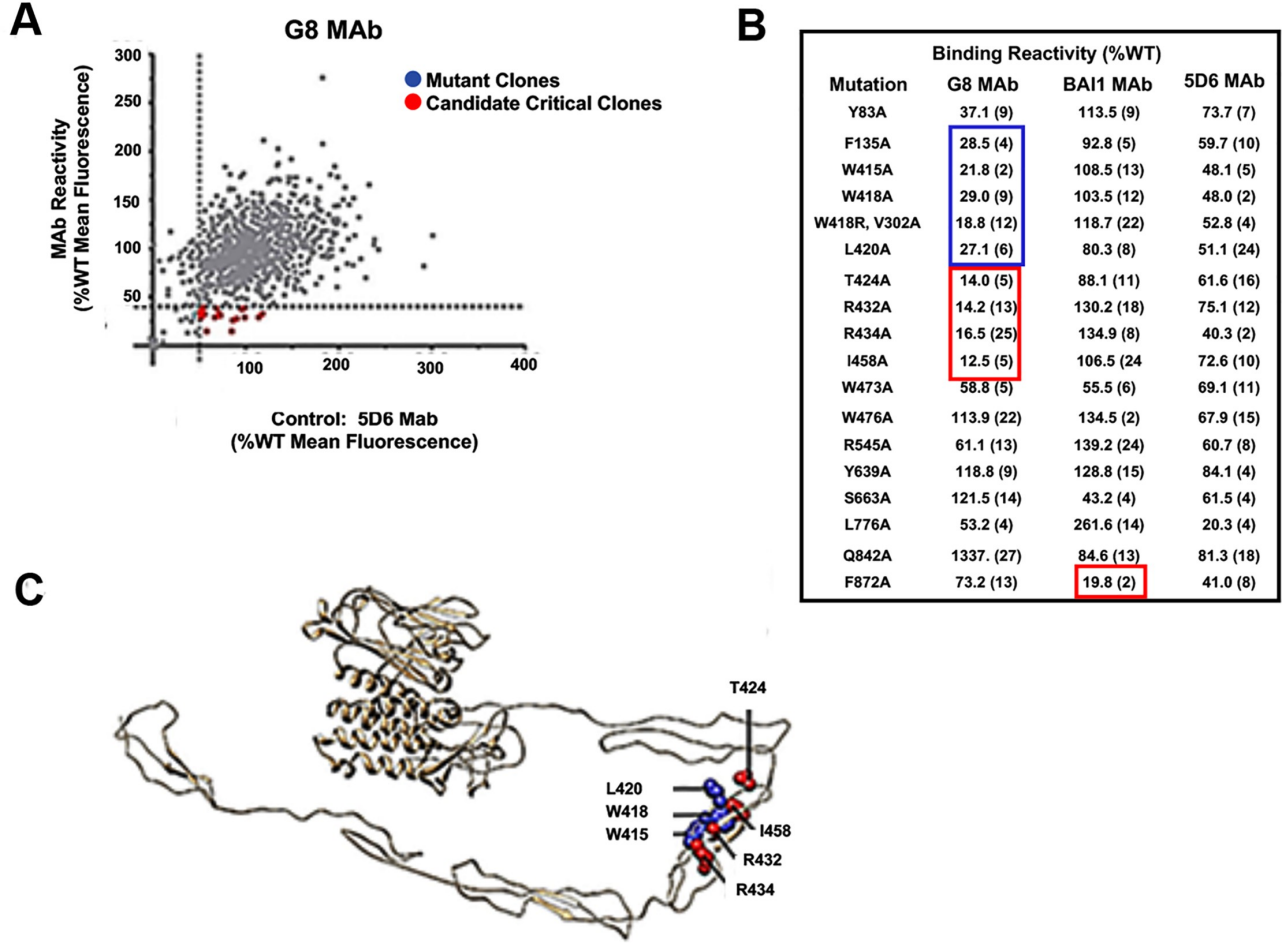
Identification of epitope of the G8 mAb. An alanine-scan library of BAI1 was constructed. The G8 and reference 5D6 mAbs were screened for binding to each individual BAI1 variant. A. Identification of candidate critical clones for G8 mAb binding was determined in duplicate by high throughput flow cytometry. For each point, background fluorescence was subtracted from the raw data that were then normalized to G8 reactivity with wild type BAI1. For each mutant clone, the mean binding value was plotted as a function of expression represented by control antibody reactivity. Candidate critical clones (red circles) were identified with a threshold (dashed line) of <40% binding of the G8 mAb and >50% of binding of the control 5D6 mAb to wild type BAI1. B. Identification of validated critical residues for G8 mAb binding. Candidate critical residues for G8 were rescreened in quadruplicate and the mean binding reactivities obtained. Validated critical residues for G8 binding (outlined in red) were residues whose mutations that resulted in <20% of wild type binding but positive (>70% binding) for binding of the 5D6 control mAb. Additional validated secondary residues (outlined in blue) were identified that did not meet the threshold guidelines but whose decreased binding activity and proximity to critical residues suggested that they may be part of the antibody epitope. C. Visualization of validated critical residues for G8 mAb binding. Critical residues (red spheres) and secondary residues (blue spheres) which may also contribute to binding were visualized on two Phyre-generated model structures that were combined to model BAI1 residues 262–934. The G8 critical residues were visualized on a model of the BAI1 thrombospondin repeat domains based on the structure of properdin (PDB ID# 1WOR; [15].

Shotgun mutagenesis was performed to individually substitute amino acids with alanine, or alanine residue with serine. Candidate critical clones expressing a single amino acid substitution were identified when G8 mAb binding was <40% of its binding to wild type BAI1 (Fig 3A). Candidate clones were rescreened four times, and those residues whose mutations reduced G8 binding by <20% of wild type BAI1 were identified as critical residues (Fig 3B). Binding of the G8 mAb was reduced to 83–87% of that of the reference anti-BAI1 mAb 5D6 (Integral Molecular) when mutations were generated in the third thrombospondin repeat of the extracellular domain of human BAI1 between amino acids 424 and 458 (Fig 3B and 3C).

### BAI1 mAbs bind to Myo/Nog cells in human tattooed skin

**Fig 4:**
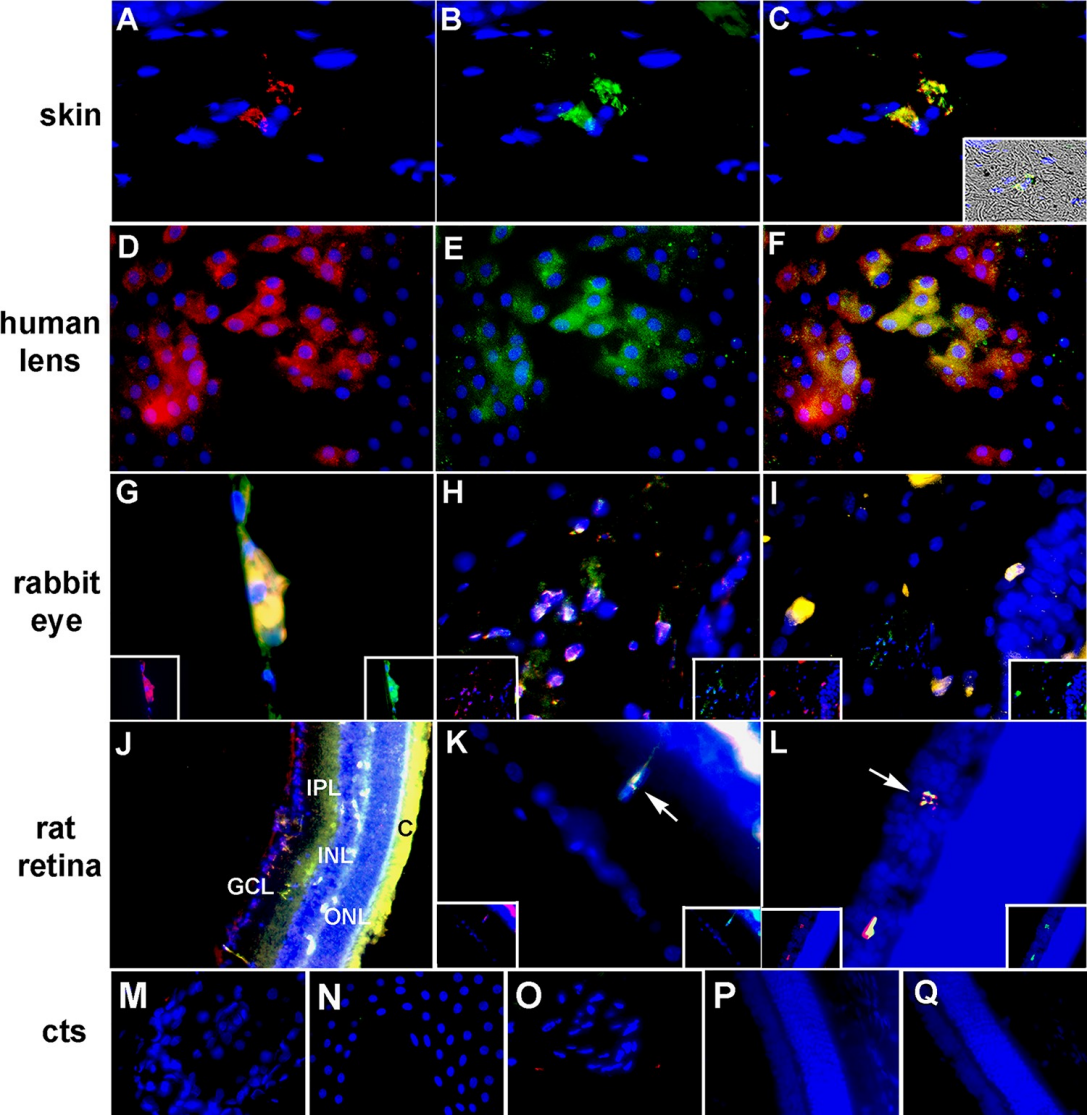
Co-localization of the G8 and R&D BAI1 mAbs in the skin and eyes. Tissue sections of human tattooed skin (A-C), human anterior lens tissue (D-F), rabbit eyes (G-I) and rat retina (J-L) were double labeled with the G8 (red) and BAI1 (green) mAbs. Nuclei were stained with Hoechst dye (blue). Unmerged images are shown in A, B, D, E and the insets in G-I and K and L. Overlap of red and green appear yellow in triple merged images in C, F, G-I, K and L. A merge of fluorescence and DIC is shown in the inset of C. The G8 and BAI1 mAbs labeled the same cells in the skin, human lens, rabbit lens (G), ciliary body (H) and cornea (I), and the inner plexiform and inner nuclear layers of the mouse retina (K and L). Minimal background fluorescence was observed in the anti-IgM and anti-IgG secondary antibody controls for the skin (M), human lens tissue (N), rabbit lens (O) and mouse retina P and Q. Bar = 9 μM in A-I and K-Q and 54 μM in J.

**Fig 5:**
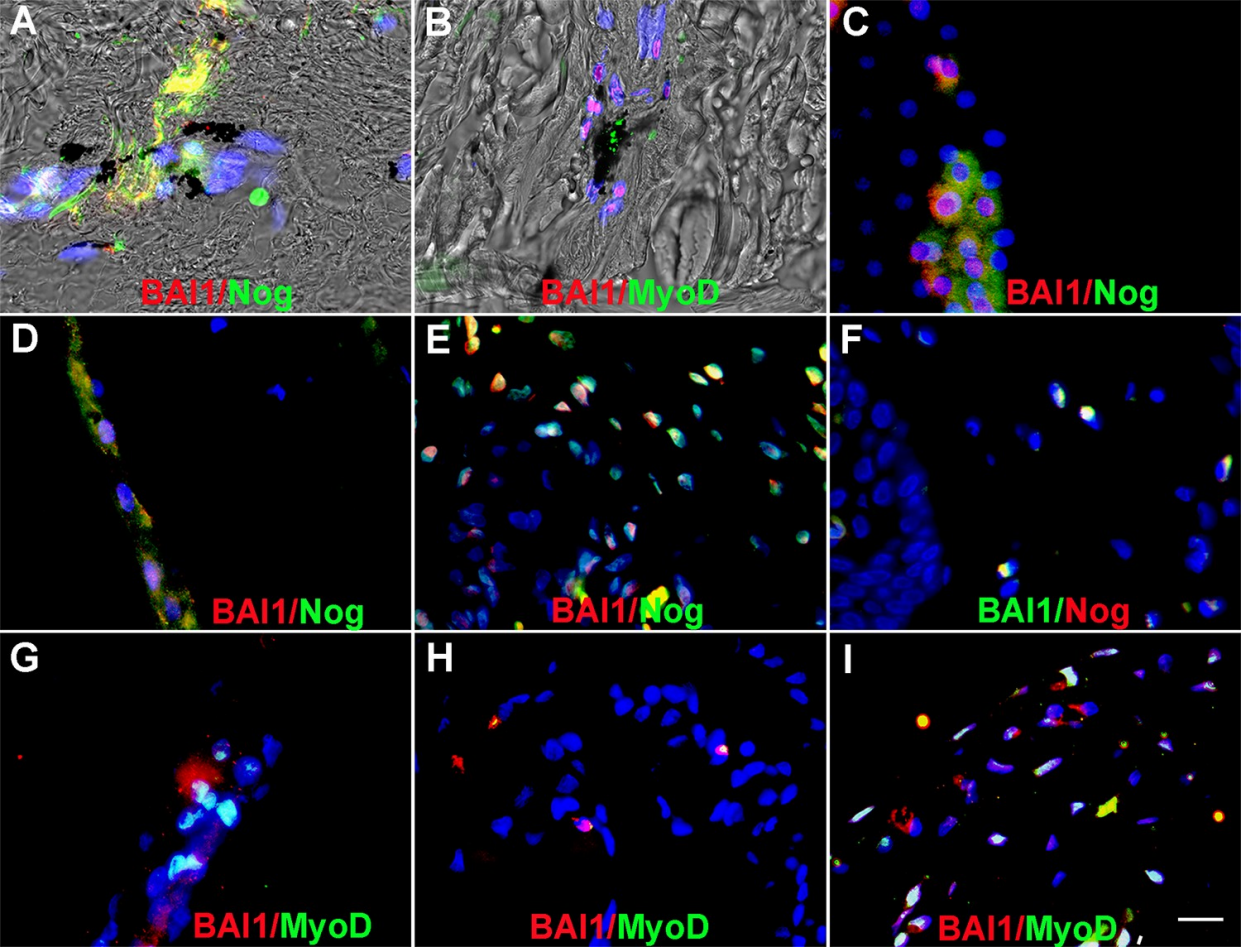
Co-localization of the R&D BAI1 mAb with antibodies to Noggin and MyoD in the skin and eyes. Tissue sections of human tattooed skin (A and B), human anterior lens tissue (C) and rabbit anterior cavity (D-I) were double labeled with the R&D BAI1 mAb and antibodies to noggin or MyoD. The colors of the fluorescent secondary antibodies are indicated in each photograph. Overlap of red and green appears yellow in merged images. Nuclei were stained with Hoechst dye. Double labeled cells were present in the skin (A and B), anterior human lens tissue (C) and the rabbit lens (D and G), ciliary body (E and H) and cornea (F and I). Bar = 9 μM.

Immunofluorescence localization was performed to compare the binding of the R&D BAI1 mAb and antibodies to G8, Noggin and MyoD in human tattooed skin. G8 co-localized with Noggin and there were no single labeled cells (Table 2). Double labeling also revealed that G8 and the R&D BAI1 mAb labeled the same cells in tattooed skin and some contained ink (Fig 4A–4C; Table 2). Few, if any G8+ cells lacked staining for BAI1 in 18 sections (Table 2). The R&D BAI1 mAb also co-localized with Noggin (Fig 5A; Table 2) and no single labeled cells were found in 12 sections.

**Table 2:** Localization of antibodies to BAI1, Noggin and MyoD in the skin, eyes and brain. Human lens tissue and tissue sections of human tattooed skin, rabbit anterior cavity, rat retina and mouse brain were double labeled with the G8 and R&D BAI1 mAbs and antibodies to Noggin and MyoD. The total numbers of double labeled cells were counted in a subset of tissue sections. The total numbers of single labeled cells were quantified in all sections with the exception of MyoD-/R&D BAI1 mAb+ and MyoD-/G8- cells that were counted in a subset of skin and anterior cavity sections. The numbers of tissues and sections scored are indicated in parenthesis. The results are the mean ± standard deviation. The R&D BAI1 mAb co-localized with G8 and Noggin with rare exception. BAI1+ cells without MyoD were present in the skin and anterior cavity.

Tissue	G8+/R&D BAI1+	G8+/R&D BAI1-	G8-/R&D BAI1+
Human tattooed skin	15 ± 4 (4)	0–3 cells (18)	0 (18)
Human lens	46 ± 14 (4)	0 (4)	0 (4)
Rabbit anterior cavity	20 ± 8 (8)	0 (14)	0 (14)
Rat retina	12 ± 1 (4)	0 (8)	0 (8)
Mouse brain	27 ± 19 (7)	0–3 cells (26)	0–7 cells (26)
	**Nog+/R&D BAI1+**	**Nog+/R&D BAI1-**	**Nog-/R&D BAI1+**
Human tattooed skin	27 ± 8 (4)	0 (12)	0 (12)
Human lens tissue	42 ± 10 (4)	0 (4)	0 (4)
Rabbit anterior cavity	16 ± 5 (4)	0 (13)	0 (13)
Rat retina	12 ± 1 (4)	0 (4)	0 (4)
Mouse brain	41 ± 31 (7)	0–3 cells (18)	0–3 cells (18)
	**G8+/Nog+**	**G8+/Nog-**	**G8-/Nog+**
Human tattooed skin	15 ± 5 (5)	0 (25)	0 (25)
	**MyoD+/R&D BAI1+**	**MyoD+/R&D BAI1-**	**MyoD-/R&D BAI1+**
Human tattooed skin	4 ± 1 (4)	0 (22)	25 ± 10 (4)
Rabbit anterior cavity	14 ± 4 (4)	0 (18)	4 ± 1 (4)
	**MyoD+/G8+**	**MyoD+/G8-**	**MyoD-/G8+**
Human tattooed skin	2 ± 1 (4)	0 (8)	12 ± 5 (4)

Labeling of tattooed skin sections with the MyoD mAb and G8 or the R&D BAI1 mAb revealed subpopulations of double and single labeled cells (Fig 5B; Table 2). All MyoD+ cells were G8+ or R&D BAI1+ (Table 2). A subpopulation of BAI1+ cells lacked MyoD (Table 2). The presence of BAI1+/MyoD- cells was expected since inactive Myo/Nog progenitor cells express MyoD mRNA but not protein and MyoD is downregulated in some G8+ cells following differentiation [2, 4, 10, 12].

### BAI1 mAbs bind to Myo/Nog cells in the eye

The anterior cavities (lens, ciliary body and cornea) of humans, rabbits and mice contain G8+/Noggin+/MyoD+ Myo/Nog cells [10–13]. In human lens tissue removed during cataract surgery, the R&D BAI1 mAb labeled the same cells as G8 (Fig 4D–4F; Table 2) and the Noggin antibody (Fig 5C; Table 2). There were no single labeled cells in this tissue (Table 2).

All R&D BAI1+ cells in the lens, ciliary body and cornea of the rabbit were labeled with G8 (Fig 4G–4I; Table 2) and the Noggin antibody (Fig 5D–5F; Table 2). Single labeled cells were not observed in any of these structures (Table 2). All MyoD+ cells were labeled with G8 and the R&D BAI1 mAb; however, a subpopulation of BAI1+ cells was negative for MyoD (Fig 4G–4I; Fig 5G–51; Table 2).

We previously reported that G8+/Noggin+/MyoD+ Myo/Nog cells are present in low numbers in the inner and outer layers of the retina and choroid of rats and mice where they were distinct from Iba1+ microglia, GFAP+ Muller glial cells, calretinin+ ganglion cells, Chx10+ bipolar cells and photoreceptors [16, 17]. Sections of the rat retina were double labeled with the R&D BAI1 mAb and the G8 or Noggin antibodies. Double labeled cells were a minor subpopulation in the inner plexiform and inner nuclear layers (Fig 4K and 4L), ganglion cell, outer plexiform and outer nuclear layers, and choroid (Table 2). Single labeled cells were not observed in this tissue (Table 2).

### BAI1 mAbs bind to Myo/Nog cells in the mouse brain

**Fig 6:**
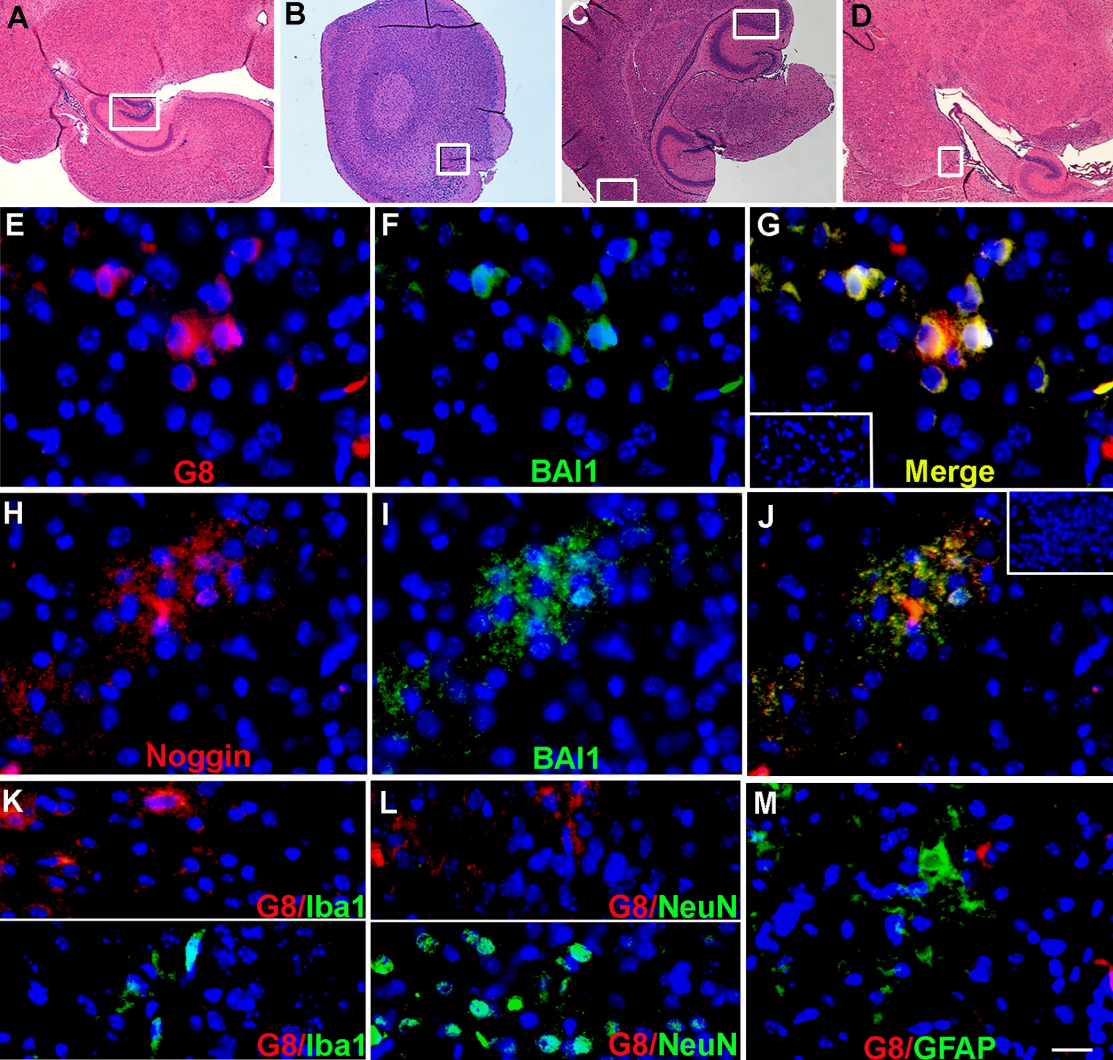
Localization of BAI1, Noggin, Iba1, NeuN and GFAP in the mouse brain. Tissue sections from the day-10 mouse brain were stained with hematoxylin and eosin (sagittal sections A and D; coronal sections B and C) or double labeled with the G8 and the R&D BAI1 mAbs, or G8 and antibodies to Iba1, NeuN or GFAP. The areas within the boxes of the H&E stained sections are shown at high magnification in the fluorescence photomicrographs. The colors of the fluorescent secondary antibodies are indicated in the unmerged photographs (E, F, H and I). Nuclei were stained with Hoechst dye. Overlap of red and green, when present, appears yellow in merged images (G, J, K, L and M). The G8 and BAI1 mAbs labeled the same subpopulation of cells in the hippocampal formation (box in A; E-G). The Noggin and BAI1 antibodies also bound to the same cells in the glomerular layer of the olfactory bulb (box in B; H-J). G8 did not co-localize with Iba1 (K, from box in A), NeuN (L from lower box in C) or GFAP (M from box in D). Minimal fluorescence was observed with the anti-IgM and anti-IgG (inset in G) or the anti-goat and anti-IgG (inset in J) secondary antibodies. Bar = 270 μM in A-D and 9 μM in E-M.

Additional double label experiments were performed to screen Myo/Nog cells for BAI1 expression in the day 10-day mouse brain that was reported to contain the highest level of BAI1 mRNA [18]. Low numbers of Myo/Nog cells were present in small clusters in the cerebral cortex and deeper structures of the brain (Fig 6). The R&D BAI1 mAb co-localized with the G8 and Noggin antibodies (Fig 6E–6J; Table 2). Double labeled cells were consistently observed in greater numbers in the hippocampal formation (Fig 6E–6G). Single labeled cells were either absent or present in very low numbers within the brain (Fig 6; Table 2). No G8+ cells contained detectable levels of the macrophage/microglia marker Iba1 (Fig 6K), the neuronal marker NeuN (Fig 6L) or GFAP (Fig 6M).

In all of these tissues, the secondary antibodies alone produced minimal background fluorescence that appeared, for the most part, as a diffuse hue (Figs 4 and 6). These double label experiments further demonstrate that the G8 mAb recognizes BAI1. With rare exception, the two BAI1 mAbs appear to be specific markers for Myo/Nog cells in these assays.

## Discussion

BAI1 is a member of the adhesion G protein-coupled receptor family [19, 20]. Originally identified as a gene target for the p53 tumor suppressor, BAI1 was reported to be expressed in neurons, astrocytes, monocytes/macrophages, vascular endothelial cells, tumors and skeletal muscle [18, 21–30]. Although expression of BAI1 in macrophages was recently called into question [31], previous detection of BAI1 protein in other lineages differs from its more restricted distribution mapped with G8 and R&D BAI1 mAbs in our experiments. Both mAbs co-localized to the same cells in the skin, eyes and brain in multiple species. Labeling with G8 or the BAI1 mAb was not detected in Iba1+ macrophages and microglia, GFAP+ glial cells in the retina and brain, or NeuN+ neurons (current study; [16, 17].

Our analyses of the chick embryo and adult mammalian tissues demonstrated that the G8 mAb is a specific marker for MyoD and noggin expressing cells [2–5, 7, 10, 11, 16, 17, 32, 33]. Furthermore, G8 did not bind to NeuroM+ neuronal precursors or neurofibrillary acid protein+ neurons [2, 5]. While more extensive screening of the G8 and R&D BAI1 mAbs in developing brains during active periods of differentiation and synaptogenesis is warranted, it is possible that the difference in our results from those illustrating a wider distribution of BAI1 reflects variations in the antigenic determinants of the antibody probes. In previous studies, BAI1 protein was detected with polyclonal antisera generated against the human extracellular domain, amino acids 103–118 [26],145–168 [22] and 601–773 [18], mouse extracellular domain, amino acids 601–773 [18] or the C-terminus [23, 24, 34]. Another polyclonal antiserum generated against the extracellular domain required tissue incubation at pH 11 for binding [24]. The critical region for G8 mAb binding includes amino acids 424–458 in the third thrombospondin repeat. This particular region may vary between cell types or is inaccessible to the G8 and R&D mAbs in non-Myo/Nog cells.

BAI1 has several functions including inhibition of angiogenesis, mediation of phagocytosis via binding of phosphatidylserine that appears on the outer leaflet of dying cells, regulation of synapse and dendritic spine formation, synthesis of reactive oxygen species, ubiquitination and levels of P53, signal transduction, cell adhesion, cytoskeleton organization and promotion of myoblast fusion [19, 21, 23, 25, 35–38]. Identification of BAI1 expression in Myo/Nog cells directs further experimentation to define their functions. For example, in addition to their essential role in regulating skeletal muscle differentiation by blocking BMP signaling [3, 6], Myo/Nog cells may be important for myoblast fusion. Comparisons of Myo/Nog cell behaviors in normal and diseased tissues of wild type and BAI1 null mice will be important for assigning specific functions to BAI1 in this lineage.
